# Configuration of Ten Light-Harvesting Chlorophyll *a*/*b* Complex I Subunits in *Chlamydomonas reinhardtii* Photosystem I^1^

**DOI:** 10.1104/pp.18.00749

**Published:** 2018-08-20

**Authors:** Shin-Ichiro Ozawa, Till Bald, Takahito Onishi, Huidan Xue, Takunori Matsumura, Ryota Kubo, Hiroko Takahashi, Michael Hippler, Yuichiro Takahashi

**Affiliations:** a Research Institute for Interdisciplinary Science, Okayama University, Okayama 700-8530, Japan; b Japan Science and Technology Agency-CREST, 4-1-8 Kawaguchi, Saitama 332-0012, Japan; c Institute of Plant Biology and Biotechnology, University of Münster, 48143 Münster, Germany

## Abstract

In plants, the photosystem I (PSI) core complex stably associates with its light-harvesting chlorophyll *a*/*b* complex I (LHCI) to form the PSI-LHCI supercomplex. The vascular plant PSI core complex associates with four distinct LHCI subunits, whereas that of the green alga *Chlamydomonas reinhardtii* binds nine distinct LHCI subunits (LHCA1–LHCA9). The stoichiometry and configuration of these LHCI subunits in the PSI-LHCI supercomplex of *C. reinhardtii* remain controversial. Here, we determined the stoichiometry of the nine distinct LHCI subunits relative to PSI subunits through uniform labeling of total proteins using ^14^C. We separated the nine LHCI polypeptides by three different sodium dodecyl sulfate-polyacrylamide gel electrophoresis systems. Our data revealed that the PSI-LHCI supercomplex contains two LHCA1 proteins and one of each of the other eight LHCI subunits. Subsequently, we identified their cross-linked products by immunodetection and mass spectrometry to determine the configuration of the 10 LHCI subunits within the PSI-LHCI supercomplex. Furthermore, analyses of PSI-LHCI complexes isolated from Δ*LHCA2* and Δ*LHCA5* mutants and oligomeric LHCI from a PSI-deficient (Δ*psaA*/*B*) mutant provided supporting evidence for the LHCI subunit configuration. In conclusion, eight LHCI subunits bind to the PSI core at the site of PSAF subunit in two layers: LHCA1-LHCA8-LHCA7-LHCA3 from PSAG to PSAK, in the inner layer, and LHCA1-LHCA4-LHCA6-LHCA5 in the outer layer. The other two LHCI subunits, LHCA2 and LHCA9, bind PSAB between PSAG and PSAH, PSAG-LHCA9-LHCA2-PSAH. Our study provides new insights into the LHCI configuration linked to the PSI core.

## Abstract (precis)

The stoichiometry and configuration of light-harvesting chlorophyll a/b complex I (LHCI) of Chlamydomonas reinhardtii reveal how 10 LHCI subunits are organized in the PSI supercomplex.

PSI catalyzes a series of light-induced electron transfer reactions from plastocyanin (or cytochrome *c*) to ferredoxin. The three-dimensional structure of PSI has been resolved at the atomic resolution in cyanobacteria (Jordan et al., 2001) and in vascular plants (Ben-Shem et al., 2003; Amunts et al., 2007, 2010; Mazor et al., 2015, 2017; Qin et al., 2015), which revealed that the structure of the PSI core complex is well conserved (Jordan et al., 2001; Ben-Shem et al., 2003; Jensen et al., 2007). The PSI core complex consists of a heterodimer of the reaction center (RC) subunits, PSAA/PSAB, as an RC, the redox components involved in the primary photochemical reactions, such as the primary electron donor, P700, and the intermediate electron acceptors, A_0_, A_1_, and F_X_, as well as core antenna pigments and several peripheral subunits. One of the peripheral subunits, PSAC, binds the secondary electron acceptors, F_A_ and F_B_, and, together with PSAD and PSAE subunits, forms a stromal ridge providing a ferredoxin docking site. PSAF and PSAN subunits are involved in plastocyanin (or cytochrome *c*) docking. In vascular plants, three hydrophobic subunits, PSAI, PSAL, and PSAH, are located on the opposite side of PSAF with respect to the PSI RC. The PSAO subunit, which has two putative transmembrane helices, also is located close to PSAL subunit and, together with PSAI, PSAL, and PSAH subunits, is proposed to be involved in the interaction with light-harvesting chlorophyll *a*/*b* complex II (LHCII; Jensen et al., 2004). In addition, the vascular plant PSI core binds the outer antenna, light-harvesting chlorophyll *a*/*b* complex I (LHCI), to form a PSI-LHCI supercomplex (Boekema et al., 2001; Ben-Shem et al., 2003). The association of LHCIs significantly increases light-harvesting capacity. The amino acid sequences, as well as secondary and tertiary structures, of the LHCI subunits are well conserved. According to the crystal structure of the vascular plant PSI-LHCI, four LHCI subunits (LHCA1–LHCA4) are located in a half-ring arrangement centered on the PSAF subunit, of which association is stabilized by the binding of PSAG and PSAK to PSAB and PSAA, respectively (Jensen et al., 2000, 2002; Moseley et al., 2002; Varotto et al., 2002; Ben-Shem et al., 2003; Ozawa et al., 2010). PSAN is peripherally associated with the luminal side of LHCA2 and LHCA3, adjacent to PSAF and PSAJ (Amunts et al., 2007). In contrast to the configuration of LHCI subunits in the plant PSI-LHCI supercomplex, the arrangement and stoichiometry of LHCI subunits in the green algal PSI complex were not sufficiently determined because of the difficulty of isolating intact green algal PSI-LHCI complexes and/or the possible presence of more LHCI subunits in the green algal complex (Busch et al., 2010; Busch and Hippler, 2011).

In the green alga *Chlamydomonas reinhardtii*, LHCI was described initially as chlorophyll protein complex O, which accumulates even in a PSI-deficient mutant (Wollman and Bennoun, 1982). It is currently accepted that the PSI-LHCI supercomplex of *C*. *reinhardtii* has nine distinct LHCI subunits (LHCA1–LHCA9; Elrad and Grossman, 2004; Takahashi et al., 2004; Stauber et al., 2009). An estimate for the stoichiometry of LHCI subunits of *C. reinhardtii* was first attempted based on the intensity of LHCI polypeptides stained with Coomassie Brilliant Blue separated by SDS-PAGE, and it was concluded that at least seven LHCI subunits per PSI are present (Bassi et al., 1992). Later, quantitative mass spectrometry was applied, which estimated nine LHCI subunits per PSI (Stauber et al., 2009; Drop et al., 2011). It was first reported that 7.5 ± 1.4 copies of LHCI subunits are present in the PSI-LHCI supercomplex isolated under photoheterotrophic growth conditions (Stauber et al., 2009). Later, it was reported that nine distinct LHCI subunits are stoichiometrically associated with the PSI core (Drop et al., 2011) when the complex was isolated from low-light photoautotrophic growth conditions. In addition, the projection maps of isolated PSI-LHCI preparations obtained by electron microscopy estimated the presence of 11 (Kargul et al., 2003), six (Kargul et al., 2005), and 14 (Germano et al., 2002) LHCI subunits. The most recent projection maps suggested the presence of nine LHCI subunits (Drop et al., 2011, 2014). Thus, the copy number of LHCI subunits in the PSI-LHCI supercomplex still remains ambiguous.

Knowledge of the configuration of the LHCI subunits in the PSI-LHCI supercomplex of *C. reinhardtii* is still limited. Two models have been proposed: in the first model, all LHCI subunits are arranged at the site of PSAF in two layers (Kargul et al., 2003; Drop et al., 2011); in the second model, a few LHCI subunits are associated with the PSI core on the opposite side of the PSAF subunit (Germano et al., 2002; Kargul et al., 2005). A more recent single-particle analysis suggested that nine LHCI subunits are arranged in two layers at the side of PSAF: four LHCI subunits are in an inner layer while five subunits are in an outer layer (Drop et al., 2011). Knowledge of the configuration of individual LHCI subunits is even more limited. One study proposed that LHCA2 and LHCA9 are located side by side and are present near PSAG (Drop et al., 2011). Other studies reported that LHCA3 is adjacent to PSAK, based on biochemical analyses of remodeling during iron deficiency (Moseley et al., 2002; Naumann et al., 2005). In the absence of chlorophyll (Chl) *b*, six LHCI subunits (LHCA1, LHCA2, LHCA3, LHCA7, LHCA8, and LHCA9) remain associated with the purified PSI subcomplex, whereas three LHCI subunits (LHCA4–LHCA6) are lost during PSI preparation, suggesting that LHCA4 to LHCA6 are present in the outer layer and, thus, are more loosely associated with the PSI core (Bujaldon et al., 2017).

Here, we report both the stoichiometry and configuration of nine distinct LHCI subunits in the PSI-LHCI supercomplex of *C. reinhardtii*. To determine the stoichiometry of the LHCI subunits, we employed uniform labeling of total cellular proteins with ^14^C, followed by separation of the nine similar LHCI polypeptides by three different SDS-PAGE systems, to estimate the amounts of PSI and LHCI subunits. Subsequently, the configuration of all LHCI subunits in the PSI-LHCI supercomplex was determined by chemical cross-linking in combination with the identification of cross-linked products by immunoblotting and mass spectrometry. The resulting structural model of LHCI subunit configuration was confirmed by the biochemical analyses of two LHCI mutants deficient in either LHCA2 or LHCA5 and a PSI-deficient mutant.

## RESULTS

### Stoichiometry of Nine Distinct LHCI Subunits in the PSI-LHCI Supercomplex

The PSI-LHCI supercomplexes of the green alga *C. reinhardtii*, which bind nine distinct LHCI subunits (LHCA1–LHCA9), usually have been isolated from cells grown photoheterotrophically in Tris-acetate-phosphate (TAP) medium (17 mM acetate; Stauber et al., 2003; Takahashi et al., 2004; Tokutsu et al., 2004). To determine the stoichiometry of the nine LHCI subunits, we grew cells in high-salt-minimal (HSM) medium supplemented with [^14^C]acetate (40 μM acetate) to uniformly label total cellular proteins with ^14^C. Thus, we isolated the PSI-LHCI supercomplexes from cells grown in medium containing 40 μm or 17 mm acetate to assess whether the concentration of acetate in the medium affected the accumulation of LHCI subunits. For this purpose, we used a small swinging-bucket rotor as described in “Materials and Methods” to make the ultracentrifugation time as short as possible, as described (Takahashi et al., 2004; Ozawa et al., 2010), because longer ultracentrifugation often detaches some unstable subunits. The apparent size of the resulting PSI-LHCI supercomplex is about 700 kD (Ozawa et al., 2010), and separation of the supercomplex by blue native (BN)-PAGE showed that the preparation was homogenous (Supplemental Fig. S1B). We confirmed that the polypeptide compositions of the two PSI-LHCI preparations isolated from the cells grown in the medium containing acetate at different concentrations were similar (Supplemental Fig. S1A), indicating that the acetate concentration in the growth medium did not substantially affect the stoichiometry of LHCI subunits in the PSI-LHCI supercomplex.

**Figure 1.**
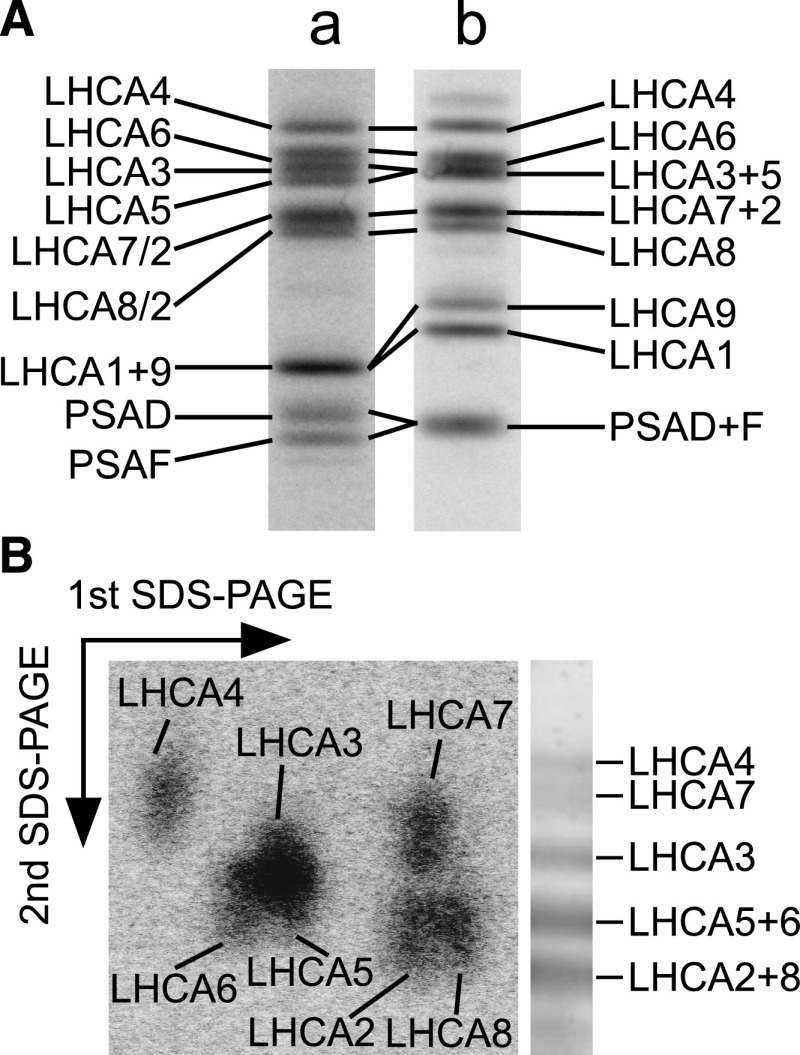
Autoradiograms of ^14^C-labeled PSI-LHCI polypeptides. A, Lane a, Separation by High-Molarity-Tris SDS-PAGE at 55°C. LHCA2 was detected in LHCA7 and LHCA8 bands, indicated as LHCA7/2 and LHCA8/2, respectively. LHCA1 and LHCA9 were detected in the same band, indicated as LHCA1+9. Lane b, Separation by High-Molarity-Tris SDS-PAGE at 6°C. LHCA1 and LHCA9 were separated as two distinct bands. LHCA3 and LHCA5 as well as LHCA7 and LHCA2 were detected in the same bands, indicated as LHCA3+5 and LHCA7+2, respectively. B, Separation by 2D-SDS-PAGE. The separation profile by the MES-Tris system is shown to the right of the 2D profile.

Since the separation of nine distinct LHCI polypeptides with similar molecular mass by a single SDS-PAGE system is difficult, we employed three different SDS-PAGE systems. High-Molarity-Tris SDS-PAGE at 55°C clearly separated four LHCI subunits (LHCA4, LHCA6, LHCA3, and LHCA5) and two PSI subunits (PSAD and PSAF), as reported previously (Takahashi et al., 2004; Tokutsu et al., 2004; Fig. 1A, lane a). The signal intensities obtained by autoradiography were divided by the carbon number of the corresponding mature LHCI and PSI subunits (Supplemental Table S1) to evaluate the relative numbers of LHCI and PSI subunits on a PSAF basis, as summarized in Table 1. Although LHCA2/LHCA7/LHCA8 and LHCA1/LHCA9 were poorly resolved, the same electrophoresis system at 6°C separated LHCA4, LHCA1, LHCA8, and LHCA9 as distinct bands (Fig. 1A, lane b). The relative abundance of LHCA1, LHCA4, and LHCA9 was estimated assuming that the amount of PSAD and PSAF (2.17 ± 0.14), which comigrated under this electrophoretic condition, is equal to the sum total of PSAD (1.17 ± 0.19) and PSAF (1 ± 0.19), which were well separated at 55°C. Finally, to evaluate the relative amounts of LHCA2, LHCA7, and LHCA8, we employed two-dimensional (2D)-SDS-PAGE that combines High-Molarity-Tris SDS-PAGE at 6°C as the first dimension and MES-Tris-Urea SDS-PAGE (Kashino et al., 2001) as the second dimension, because the MES-Tris-Urea electrophoresis system clearly separated LHCA7 from LHCA2 and LHCA8 (Fig. 1B). This 2D-SDS-PAGE sufficiently separated these three LHCI polypeptides (Fig. 1B) and allowed calculation of the amount of LHCA2, LHCA7, and LHCA8 on a PSAF basis by dividing the total number of these three LHCA subunits evaluated by High-Molarity-Tris SDS-PAGE at 55°C by the ratios obtained by 2D-SDS-PAGE. In conclusion, the PSI-LHCI supercomplex contained approximately two copies of LHCA1 (1.81 ± 0.07) and nearly one copy of each of the other LHCI subunits, indicating that the supercomplex contains a total of 10 LHCI subunits.

**Table 1.**
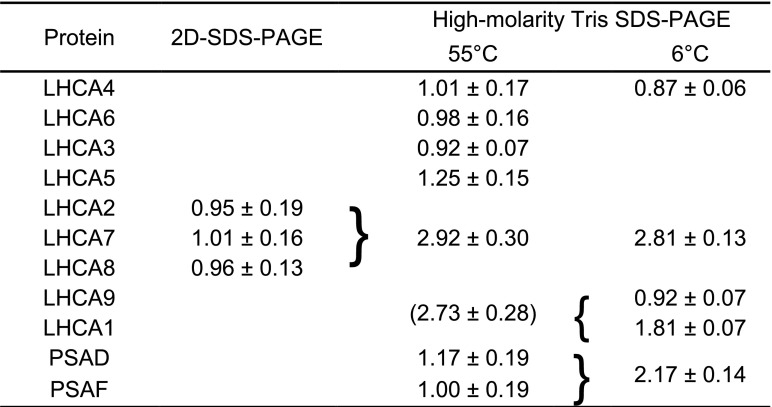
Relative abundance of LHCI and PSI subunits in PSI-LHCI The relative abundances of nine LHCI (LHCA1–LHCA9) and two PSI (PSAD and PSAF) subunits were estimated. Subunits are listed according to the order of migration in High-Molarity-Tris SDS-PAGE at 55°C. The relative abundances of LHCA4, LHCA6, LHCA3, LHCA5, PSAD, and PSAF were determined by High-Molarity-Tris SDS-PAGE at 55°C, those of LHCA4, LHCA9, and LHCA1 by High-Molarity-Tris SDS-PAGE at 6°C, and those of LHCA2, LHCA7, and LHCA8 by 2D-SDS-PAGE. The values are normalized against PSAF and presented as means ± se across three biological replicates.

### Chemical Cross-Linking of the PSI-LHCI Subunits

**Figure 2.**
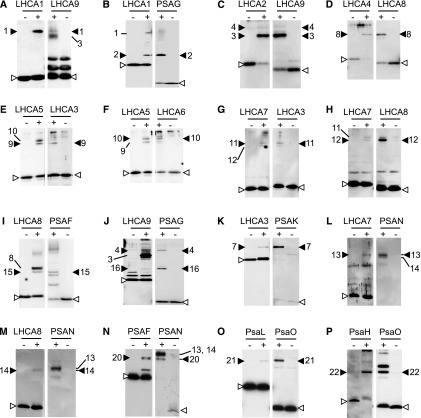
Immunochemical identification of cross-linked products of PSI and LHCI subunits. Cross-linked products of PSI and LHCI subunits were separated and identified by immunoblotting using a battery of antibodies against each of the PSI and LHCI subunits. Two nitrocellulose membrane strips were incubated separately with each of the two antibodies indicated at the top of each pair of gels. Polypeptides before (−) and after (+) chemical cross-linking were subjected to SDS-PAGE. The chemical cross-linkers used were DSG (A and B), DSS (C–K), EDC (L–N), and DMS (O and P), as summarized in Table 2. Black arrowheads indicate cross-linked products, whereas white arrowheads correspond to non-cross-linked products. The cross-linked product identification numbers are as shown in Table 2. The bands assigned as 13 and 14 correspond to the cross-linked products of LHCA7/PSAN and LHCA8/PSAN, respectively.

**Figure 3.**
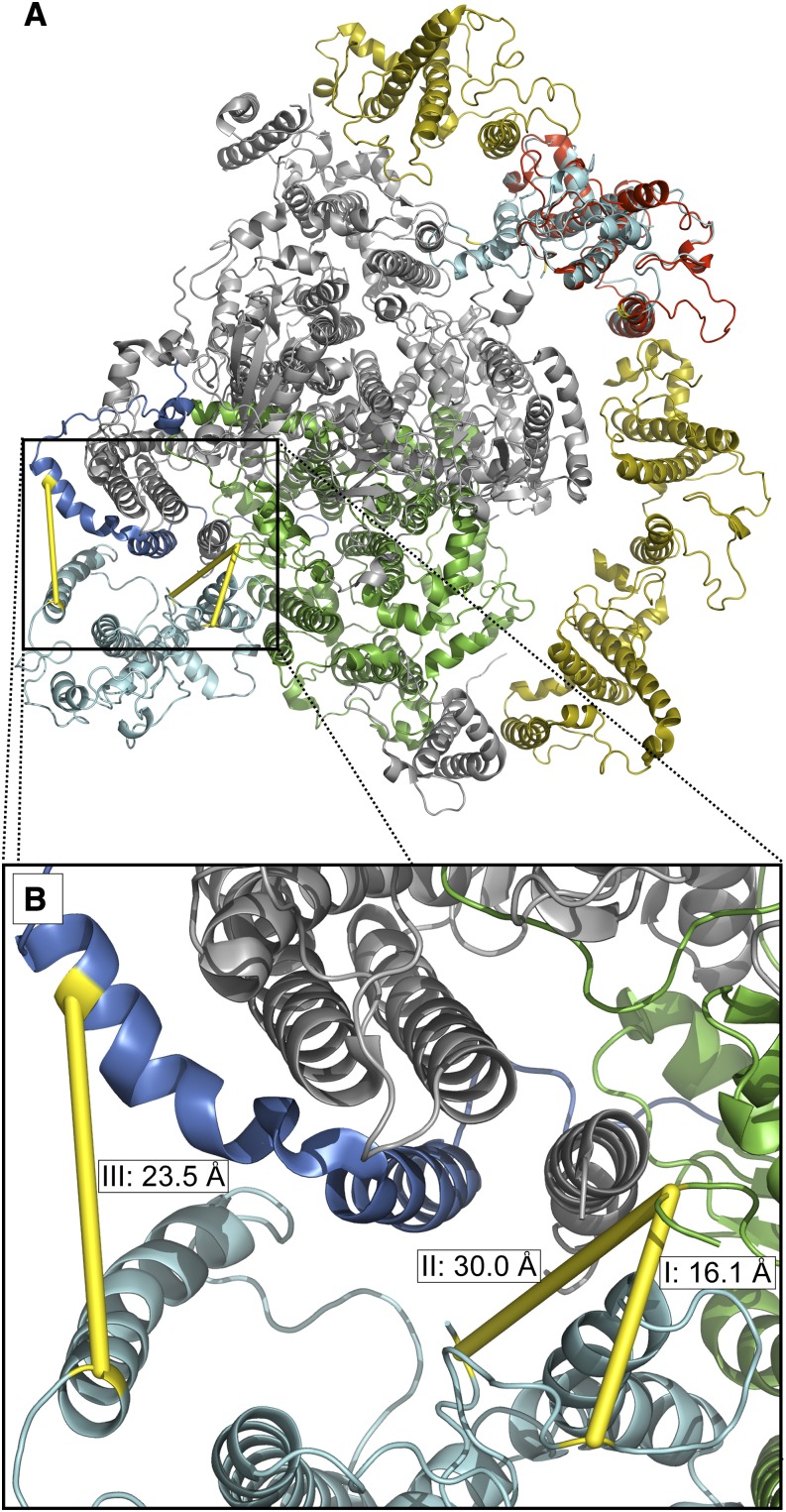
LHCA2 position at the PSAH side of the PSI core. The PSI-LHCI structure from vascular plants (protein database identifier 4Y28) is shown as an overview (A) and a closeup of the cross-linked region (B), as viewed from the stromal side. The PSI core is depicted in gray, except PSAB (green) and PSAH (blue). The single light-harvesting layer of vascular plants is depicted in dark yellow, except for LHCA2 (red). Modeled *C. reinhardtii* LHCA2 (light blue) at the PSAH side of the core is in a position where the cross-link distance constraints are fulfilled (for details, see “Materials and Methods: Mapping Cross-Links to Molecular Structure”). The cross-linked peptide combinations marked with I, II, and III in Supplemental Table S2 are visualized as three different cross-links: LHCA2 with PSAB (I and II) and LHCA2 with PSAH (III). Cross-linked amino acids are highlighted in yellow, as well as their connection through the cross-linker. The cross-links were identified from LC-MS/MS data with Crosslinx.

To determine the configuration of the 10 LHCI subunits in PSI-LHCI, we performed nearest neighbor analyses of PSI and LHCI subunits. Subunits were cross-linked by incubating the isolated thylakoid membranes or the PSI-LHCI supercomplex preparations with different chemical cross-linkers [1-ethyl-3-(3-dimethylamino-propyl)-carbodiimide (EDC), disuccinimidyl suberate (DSS), disuccinimidyl glutarate (DSG), and dimethyl suberimidate (DMS)]. The resulting cross-linked products were separated by SDS-PAGE and identified by immunoblotting using a battery of specific antibodies against each of the LHCI and PSI subunits (Fig. 2). We identified 17 cross-linked products between LHCI subunits, PSI subunits, or LHCI and PSI subunits (Table 2). Cross-linked products also were subjected to liquid chromatography coupled to tandem mass spectrometry (LC-MS/MS). To improve MS/MS data analysis and identify cross-links between two peptides, a newly devised algorithm named Crosslinx was employed in two steps, an initial search against a small database and a validation search against the whole proteome, as described in Supplemental Figure S2. Finally, MS/MS data analysis identified 38 cross-linked peptide pairs belonging to the PSI-LHCI supercomplex, of which 13 were intermolecular (cross-links between different proteins; Table 2; Supplemental Table S2). Having the identified peptide sequences of LHCA2, which cross-linked independently to peptides of PSAB and PSAH, a molecular model of their interaction can be drawn (Fig. 3), as performed previously (Merkley et al., 2014). This model confirmed that all distance constraints defined by the cross-linker could be fulfilled (Fig. 3B) if LHCA2 is associated with PSAB and PSAH on the corresponding side of the PSI core. Altogether, we found 19 subunits (all nine LHCI subunits and 10 PSI subunits) in 22 cross-linked proteins/peptides (Table 2). Notably, we identified a PSAK-LHCA3 cross-link by MS/MS, as identified before in barley (*Hordeum vulgare*) PSI (Jansson et al., 1996) and herein by immunoblotting (Table 2), further underpinning the suitability of the MS approach.

**Table 2.** Detected cross-linked subunits of PSI-LHCI Cross-linked products were assigned 1 to 22 as identification numbers; apparent molecular masses of the products are shown. Cross-linked products were identified by immunoblotting (I) or mass spectrometry (M). Cross-linked product 2 was detected in the PSI-LHCI preparation, while products 11 and 12 were detected in thylakoid membranes washed with NaBr. n.d.:not determined.

No.	Cross-Linked Product	Apparent Molecular Mass [kD]	Cross-Linkers	Assignment Methods
				
1	LHCA1 + LHCA9	42	DSS, DSG	I
2	LHCA1 + PSAG	31	DSG	I
3	LHCA2 + LHCA9	43	DSS, DSG	I
4	LHCA2 + LHCA9 + PSAG	53	DSS	I
5	LHCA2 + PSAH	n.d.	DSS	M
6	LHCA2 + PSAB	n.d.	DSS	M
7	LHCA3 + PSAK	32	DSS	I, M
8	LHCA4 + LHCA8	48	DSS, DSG	I, M
9	LHCA5 + LHCA3	47	DSS	I
10	LHCA5 + LHCA6	47	DSS, DSG	I
11	LHCA3 + LHCA7	46	DSS	I
12	LHCA7 + LHCA8	44	DSS	I
13	LHCA7 + PSAN	31	EDC	I
14	LHCA8 + PSAN	31	EDC	I
15	LHCA8 + PSAF	40	DSS, DSG	I
16	LHCA9 + PSAG	31	DSS, EDC	I
17	PSAA + PSAF	n.d.	DSS	M
18	PSAB + PSAD	n.d.	DSS	M
19	PSAD + PSAF	n.d.	DSS	M
20	PSAF + PSAN	27	EDC	I
21	PSAL + PSAO	22	EDC, DSG	I
22	PSAH + PSAO	20	DMS	I

**Figure 4.**
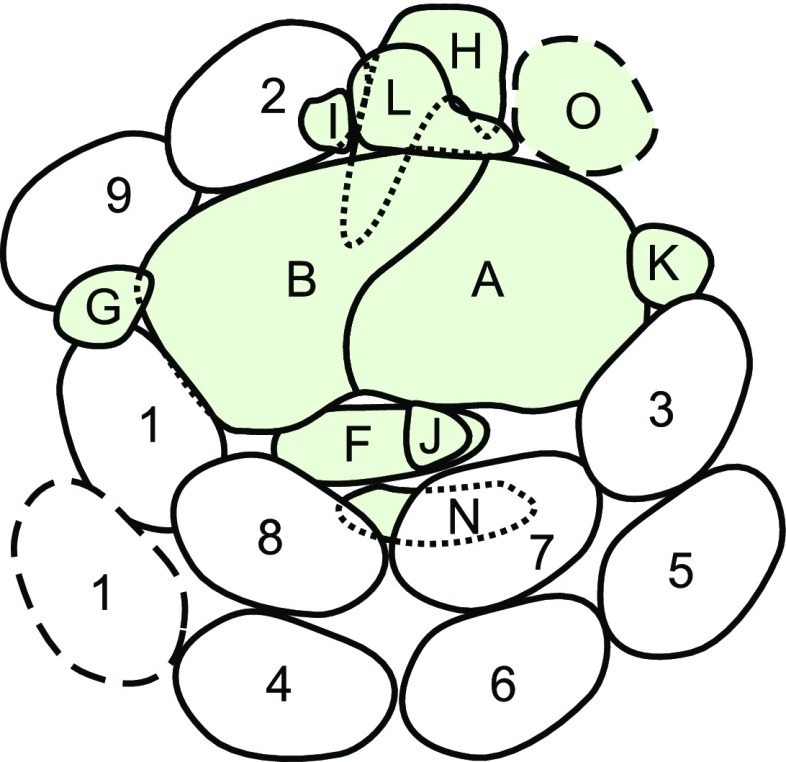
A model of the LHCI subunit configuration in PSI-LHCI. The configuration of the subunits of PSI (green) and LHCI in PSI-LHCI is viewed from the stromal side. Outlines of subunits on the lumenal side are shown with dotted lines (PSAN and PSAH). The stromal extrinsic subunits, PSAC, PSAD, and PSAE, are omitted. The locations of the second copy of LHCA1 and PSAO are drawn with dashed lines. Letters and numbers represent the names of PSI and LHCI subunits, respectively.

To put the cross-linking results together, we derived an adjacency relationship by connecting the cross-linked subunits by lines (Supplemental Fig. S3). To determine the configuration of the LHCI subunits in the PSI-LHCI, we also referred to the previously proposed structures of the plant PSI-LHCI obtained by crystallography (Ben-Shem et al., 2003; Amunts et al., 2007, 2010; Mazor et al., 2015; Qin et al., 2015) and the structure of *C. reinhardtii* PSI-LHCI, in which LHCI subunits are organized as two layers (inner and outer layer), proposed by single-particle analysis (Drop et al., 2011). The resulting adjacency relationship indicated that six LHCI subunits (LHCA1, LHCA2, LHCA3, LHCA7, LHCA8, and LHCA9) were cross-linked to one of the PSI core subunits. In the structure of the vascular plant PSI-LHCI supercomplex, four LHCI subunits are associated with the PSI core at the PSAF side (in the order PSAG, Lhca1, Lhca4, Lhca2, Lhca3, PSAK); therefore, we deduced that LHCA1, LHCA8, LHCA7, and LHCA3 are located at the corresponding sites of the *C. reinhardtii* PSI core in the inner layer, as shown in Figure 4. In contrast, our cross-linking data indicate that LHCA2 and LHCA9 lie side by side and associate with PSAB between PSAH and PSAG. The remaining four LHCI subunits, which include the other copy of LHCA1, could be located in the outer layer of LHCI subunits at the PSAF site, based on the *C. reinhardtii* PSI-LHCI structure proposed by single-particle analyses. Since LHCA4 was cross-linked with LHCA8 and LHCA5 was cross-linked with LHCA3 and LHCA6 (Fig. 2), it is likely that LHCA4, LHCA6, and LHCA5 are located in the outer layer, as shown in Figure 4. We tentatively assigned LHCA1 to the open space next to LHCA4 in the outer layer, although no cross-linking evidence was obtained. In summary, we propose that four LHCI subunits (LHCA1-LHCA8-LHCA7-LHCA3) are located in the inner layer and four LHCI subunits (LHCA1-LHCA4-LHC6-LHCA5) are located in the outer layer, at the PSAF site of the PSI core, whereas two LHCI subunits (LHCA9-LHCA2) associate with PSAB.

### Effects of the Absence of LHCA2 or LHCA5 on the Stability of LHCI Subunits

**Figure 5.**
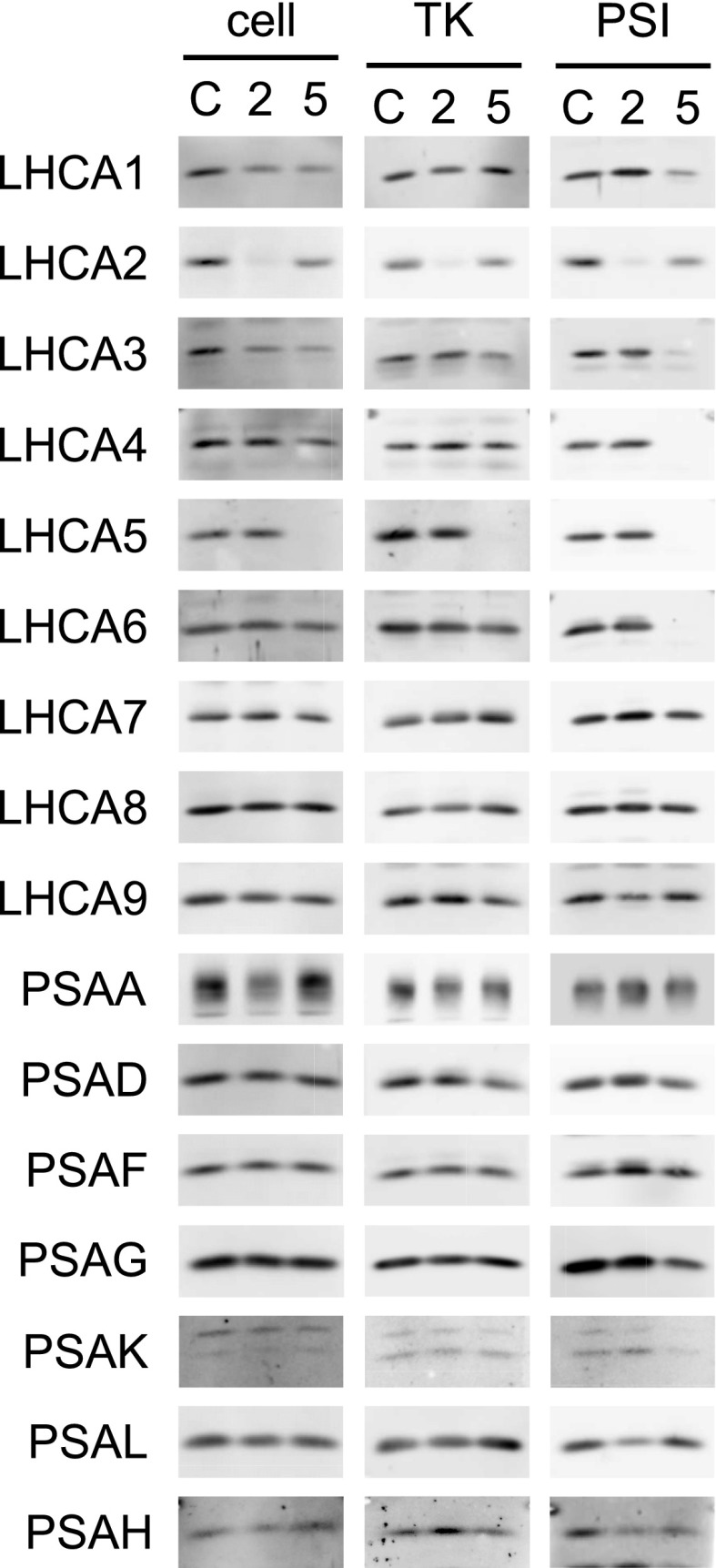
LHCI and PSI proteins of Δ*LHCA2* and Δ*LHCA5* mutants. Total cellular proteins (cell; 1 μg of Chl), thylakoid proteins (TK; 0.5 μg of Chl), and PSI-LHCI proteins (PSI) from control (C), Δ*LHCA2* (2), and Δ*LHCA5* (5) strains were separated and detected immunochemically with antibodies against LHCI subunits and PSI subunits. The loading amount for the PSI-LHCI preparations was normalized to the signal intensity of PSAD. Although faint signals were detected by anti-LHCA2 antibody in proteins from the cell, TK, and PSI from the Δ*LHCA2* mutant, they are ascribed to a cross reaction with LHCA8, which comigrates with LHCA2 under this electrophoretic condition.

To confirm the configuration of LHCI subunits in the proposed model (Fig. 4), we analyzed the contents of LHCI and PSI subunits in the cell, thylakoids, and PSI-LHCI supercomplex obtained from Δ*LHCA2* and Δ*LHCA5* mutants generated by the *Chlamydomonas* Library Project group (Li et al., 2016; Supplemental Fig. S4). The Δ*LHCA2* and Δ*LHCA5* mutants specifically lacked LHCA2 and LHCA5, respectively, and accumulated PSI subunits and the other LHCI subunits at normal levels in the cell and isolated thylakoids (Fig. 5). Then, we isolated PSI-LHCI supercomplexes from the thylakoid extracts of Δ*LHCA2* and Δ*LHCA5* mutants by sucrose density gradient ultracentrifugation, and their absorption and fluorescence spectra are shown in Supplemental Figure S5C. Interestingly, the PSI-LHCI supercomplex isolated from the Δ*LHCA5* mutant lost LHCA4 and LHCA6 and contained a reduced amount of LHCA1 (approximately 50% of the control level). Thus, LHCI subunits in the outer layer are destabilized in the absence of LHCA5, supporting the location of one of the two copies of LHCA1 as well as LHCA4 and LHCA6 in the outer layer of the LHCI subunits. The PSI-LHCI supercomplex isolated from the Δ*LHCA2* mutant retained the eight LHCI subunits located in the inner and outer layer at control levels but contained a reduced amount of LHCA9. In addition, the amount of PSAH and PSAL was decreased by approximately 50% (Fig. 5). These observations support the location of LHCA2 in proximity to PSAH, PSAL, and LHCA9, as proposed in Figure 4.

### Biochemical Evidence for the Configuration of the LHCI Subunits

We isolated a PSI-LHCI subcomplex from the wild-type thylakoids solubilized by a three-step detergent treatment (Supplemental Fig. S5). The resulting PSI preparation retained PSAL, LHCA2, and LHCA9 but lost the other LHCI subunits, PSAF, PSAG, and PSAK (Supplemental Fig. S5B). Further separation of the subcomplex by size-exclusion column chromatography revealed the presence of two peaks with apparent sizes of 340 and 270 kD (Supplemental Fig. S5C, a and b). The larger complex was a PSI-LHCI subcomplex containing LHCA2 and LHCA9 (PSI-LHCA2-LHCA9), while the smaller complex was a PSI core subcomplex. The Chl *a*/*b* ratio of PSI-LHCA2-LHCA9 was approximately 11 to 13. The absorption spectrum confirmed the lower Chl *b* and carotenoid contents of PSI-LHCA2-LHCA9 (Supplemental Fig. S5C, c). The fluorescent emission spectrum at 77K showed a peak at 716 nm, which is similar to that of PSI-LHCI (Supplemental Fig. S5C, d). These results indicate that LHCA2 and LHCA9 stably bind to the PSI core even in the absence of the other LHCI subunits. Additionally, we purified an oligomeric LHCI complex from a PSI-deficient strain (Δ*psaA/B*), as shown in Supplemental Figure S6. This purified LHCI preparation lacked LHCA2 and LHCA9 but retained the other LHCI subunits except for LHCA3, the amount of which was decreased significantly, as reported previously (Takahashi et al., 2004). This observation is consistent with the LHCI configuration model, in which LHCA2 and LHCA9 are not connected directly with the other LHCI subunits but are associated directly with the PSI core. The decreased amount of LHCA3 can be ascribed to its location on the edge of the oligomeric structure of eight LHCI subunits, as shown in Figure 4 and as reported previously (Takahashi et al., 2004).

## DISCUSSION

In this study, our data revealed that 10 LHCI subunits are present in the PSI-LHCI supercomplex of *C. reinhardtii*, as assessed by uniform labeling of the PSI-LHCI subunits with ^14^C and subsequent separation of nine distinct LHCI subunits by three SDS-PAGE systems. In addition, we used chemical cross-linking experiments and subsequent identification of the cross-linked products by immunoblotting using specific antibodies and mass spectrometry with improved MS/MS data analysis to determine the full configuration of individual LHCI subunits in the PSI-LHCI supercomplex.

### Ten LHCI Subunits Are Present in the PSI-LHCI Supercomplex

The presence of four LHCI subunits (LHCA1–LHCA4) in the vascular plant PSI-LHCI supercomplex has been demonstrated via high-resolution crystal structures (Amunts et al., 2007; Mazor et al., 2015, 2017; Qin et al., 2015). Structural information at high resolution is not available for the PSI-LHCI supercomplex of *C. reinhardtii*, but the presence of nine distinct LHCI subunits (LHCA1–LHCA9) has been shown biochemically (Elrad and Grossman, 2004; Takahashi et al., 2004; Stauber et al., 2009). However, the stoichiometry of the LHCI subunits in the algal complex has not been determined.

Based on the projection maps by electron microscopy at low resolution, LHCI subunits are organized on one side of the PSI core complex in a double half-ring arrangement with different numbers of LHCI subunits (Germano et al., 2002; Kargul et al., 2003; Drop et al., 2011, 2014). Germano et al. (2002) reported that 14 LHCI subunits could be accommodated in the PSI-LHCI supercomplex, such that two or three of the LHCI subunits bind at the side of the PSAH subunit. Kargul et al. (2003) estimated that 11 LHCA subunits are present along the side of the PSI core complex where PSAK, PSAJ, PSAF, and PSAG are located. In contrast, it was reported that the PSI-LHCI supercomplex isolated from cells locked in state 2 contains six LHCI subunits, such that four LHCI subunits are arranged in one layer while two LHCI subunits are present near PSAH/PSAL/PSAI (Kargul et al., 2005). More recently, Drop et al. (2011) reported that nine LHCI subunits are arranged in two layers at one side of the PSI core complex along with the PSAF site: four of the LHCI subunits were located in the inner layer, whereas the remaining five LHCI subunits were present in the outer layer. The localization of the nine LHCI subunits in PSI-LHCI was determined by overlapping the projection map with the model of the high-resolution structure of plant PSI-LHCI (protein database identifier 2WSC; Amunts et al., 2010). However, the most recent structure of the plant complex (protein database identifier 5L8R) is slightly compact, because some electron densities in the peripheral regions of Lhca3, PSAK, PSAA, and PSAH are absent (Mazor et al., 2017). This more-compact structure may render the configuration of the nine LHCI subunits ambiguous. Thus, the number of LHCI subunits estimated by the projection map remains controversial. The first attempts to estimate the stoichiometry of the nine LCHI subunits by mass spectrometry resulted in 7.5 ± 1.4 LHCIs (Stauber et al., 2009) and nine distinct LHCIs (Drop et al., 2011). The discrepancy in the number of LHCI subunits associated with PSI-LHCI may be ascribed to the fact that some LHCI subunits are lost from PSI preparations during solubilization and/or purification. Our ^14^C approach for the quantitative determination of LHCI subunit stoichiometry revealed that the PSI-LHCI supercomplex contains one copy of each of eight distinct LHCI subunits (LHCA2–LHCA9) as well as two copies of LHCA1. Consistently, a stronger staining of the LHCA1 polypeptide compared with that of the other LHCI polypeptides was observed on acrylamide gels in the PSI-LHCI preparation (Supplemental Fig. S1). In line with these findings, we concluded that the *C. reinhardtii* PSI-LHCI supercomplex possesses 10 LHCI subunits.

### Eight LHCI Subunits Are Arranged in Two Layers along PSAG, PSAF, PSAJ, and PSAK of the PSI-LHCI Supercomplex

Single-particle analysis previously proposed that the *C. reinhardtii* PSI-LHCI supercomplex associates with two layers of the LHCI subunits (Germano et al., 2002; Kargul et al., 2003; Drop et al., 2011); however, those works did not fully resolve the configuration of the individual LHCI subunits within the PSI-LHCI supercomplex. Our model proposes, based on cross-linking results (Table 2), that four of the LHCI subunits, LHCA1-LHCA8-LHCA7-LHCA3, are present in the inner layer of PSI-LHCI like those in plant PSI-LHCI (Fig. 4). The location of LHCA3 in proximity to PSAK is consistent with the results reported previously (Moseley et al., 2002).

Interestingly, characterization of the polypeptide composition of several PSI-LHCI subcomplexes revealed that the association of LHCA1, LHCA7, LHCA8, and LHCA3 is more stable than that of LHCA4, LHCA5, and LHCA6, indicating that the former four LHCI subunits are located in the inner layer (Drop et al., 2011). Recent analyses of the LHC subunits in a Chl *b*-deficient mutant of *C. reinhardtii*, BF3, revealed that nine LHCI subunits accumulate at wild-type levels in cells grown in moderate light, whereas only LHCA2, LHCA3, LHCA7, LHCA8, and LHCA9 remain associated with the isolated PSI complex at substantial levels (Bujaldon et al., 2017). These observations suggested that these LHCI subunits bind directly to the PSI core complex and, thus, are present in the inner layer because the stability of the configuration of LHCI subunits is impaired in the absence of Chl *b*. Our study showed that three LHCI subunits, LHCA4, LHCA5, and LHCA6, were cross-linked to other LHCI subunits but not to PSI subunits; therefore, we proposed that these LHCI subunits are located in the outer layer. The accumulation of LHCA4 and LHCA6 decreased specifically in BF3 cells grown in dim light (Bujaldon et al., 2017). Assuming that LHCI subunits in the outer layer are more susceptible to proteinase digestion, our observation in BF3 is consistent with our model that LHCA4 and LHCA6 are present in the outer layer. Based on the chemical cross-linked products, LHCA4 and LHCA5 are located in proximity to LHCA8 and LHCA3, respectively; this observation suggests that LHCA6, which lies adjacent to LHCA5, is located between LHCA4 and LHCA5. Thus, it is possible that one of the two copies of the LHCA1 subunit is accommodated in a space located in proximity to LHCA1, LHCA8, and LHCA4, suggesting that LHCA1 subunits are present in both the inner and outer layers at the PSAG side of the PSI core. This localization of LHCA1 subunits is supported by the presence of two copies of LHCA1 in the oligomeric LHCI complex isolated from the PSI-deficient mutant (Supplemental Fig. S6B, b), the reduction of one of the two LHCA1 subunits in the PSI-LHCI subcomplex isolated from the Δ*LHCA5* mutant (Fig. 5), and the result that one of the PSI-LHCI subcomplexes with significantly reduced levels of LHCA4, LHCA5, and LHCA6 also contained a reduced amount of LHCA1 (Drop et al., 2011). Collectively, these data imply that the presence of a double half-ring arrangement, with each half-ring consisting of a four-LHCI subunit, suggests that the four-LHCI subunit half-ring arrangement in the vascular plant PSI-LHCI might be duplicated in the green alga to increase the antenna size. However, the structural similarity of LHCI subunits between the two layers was not obvious, except for LHCA1 in both layers.

### LHCA2 and LHCA9 Are Located between PSAH and PSAG in the PSI Core Complex

Identification of the cross-linked peptides by mass spectrometry revealed that LHCA2 binds to PSAH and PSAB, indicating that LHCA2 is located at a site away from the eight LHCI subunits in the two layers at the PSAF site (Fig. 3). The cross-links between LHCA2 and LHCA9 indicate that LHCA2 and LHCA9 also are located side by side, which is strongly supported by biochemical analysis of the PSI-LHCI subcomplex isolated from the Δ*LHCA2* mutant, in which the amounts of LHCA9, PSAH, and PSAL were partially reduced (Fig. 5). In addition, the absence of LHCA2 and LHCA9 from the oligomeric LHCI complex isolated from the ΔPSI mutant confirmed that these two LHCI subunits are not directly or stably associated with the other LHCI subunits (Supplemental Fig. S6). Although it is reported that LHCA2 and LHCA9 are bound loosely to PSI-LHCI (Drop et al., 2011), we nevertheless were able to isolate a PSI-LHCI subcomplex associated only with LHCA2 and LHCA9 from wild-type thylakoids (Supplemental Fig. S5). These observations are in agreement with the proposed model that LHCA2 and LHCA9 are located at the side of PSAL and PSAG of the PSI core complex. Since LHCA9 was cross-linked to PSAG and LHCA1, LHCA2 is inferred to be located next to PSAH while LHCA9 is in proximity to PSAG. It also is likely that one LHCA1 subunit, which is in either the inner or outer layer, interacts with LHCA9. Furthermore, the direct binding of LHCA2 and LHCA9 to the PSI core is supported by the biochemical study of the Chl *b*-less mutant, BF3, in which LHCA2 and LHCA9 associate with the isolated PSI-LHCI subcomplex as stably as other LHCI subunits in the inner layer (Bujaldon et al., 2017). Therefore, we proposed that LHCA2 and LHCA9 subunits, which associate with low-energy chlorophylls (red chlorophylls; Mozzo et al., 2010; Le Quiniou et al., 2015), bind directly to the PSI core complex at the side of PSAL and PSAG (Fig. 4; Stauber et al., 2009). Interestingly, both LHCA2 and LHCA9 are close paralogs of green alga-specific LHCA proteins and likely coordinate low-energy chlorophylls (Stauber et al., 2009; Mozzo et al., 2010). This further strengthens the idea that the structural feature of the *C*. *reinhardtii* PSI-LHCI formed by these two polypeptides is green alga specific and, therefore, absent from vascular plants. Our model for the configuration of 10 LHCI subunits is not consistent with the model proposed by the most recent projection map of PSI-LHCI, in which four and five LHCI subunits are located in the inner and outer layers, respectively (Drop et al., 2011). As discussed above, this contradiction could be derived from the fact that the plant PSI-LHCI structure (protein database identifier 2WSC; Amunts et al., 2010) used to localize LHCI subunits still contains some additional electron densities in the peripheral regions compared with that of the more recent structure (protein database identifier 5L8R; Mazor et al., 2017).

The red alga *Cyanidioschyzon merolae*, which lacks Chl *b*, contains a PSI-LHCR supercomplex in which the PSI core associates with three distinct red algal light-harvesting complexes (LHCRs). Recently, the structures of PSI-LHCR supercomplexes have been determined by cryoelectron microscopy (Pi et al., 2018) and x-ray crystallography (Antoshvili et al., 2018). One of the PSI-LHCR supercomplexes contains three distinct LHCR subunits (LHCR1–LHCR3) at the PSAF side of the PSI core. The location of the three LHCR subunits is similar to that of LHCA4, LHCA2, and LHCA3 subunits in the vascular plant complex. Intriguingly, cryoelectron microscopy also determined the other structure in which two additional LHCR subunits, LHCR1 and LHCR2, are associated with the PSI core at the PSAH, PSAI, PSAL, and PSAX sites (Pi et al., 2018). The locations of LHCR1 and LHCR2 appear to correspond to those of LHCA2 and LHCA9, respectively, in *C. reinhardtii*. In conclusion, the configuration of LHCA2 and LHCA9 in the PSI-LHCI supercomplex also is conserved in PSI-LHCR of the red alga, supporting the view that this is an ancient form of PSI-LHCI. Interestingly, Drop et al. (2014) reported the projection map of a PSI-LHCI-LHCII particle by solubilizing the thylakoid membranes from *C. reinhardtii* cells locked in state 2 with a mild detergent. Within the 2D projection map, in addition to a PSI core and nine LHCI subunits, two LHCII trimers were located at the PSAH-PSAI-PSAL ridge. Of note, a density near PSAG and PSAH/PSAI/PSAL, which was tentatively assigned for a minor LHCII, CP29, appears to be sufficient for LHCA2 and LHCA9.

## CONCLUSION

We have determined the stoichiometry and detailed configuration of the LHCI subunits in a PSI-LHCI supercomplex isolated from *C. reinhardtii*. The stoichiometric analysis of nine distinct LHCI subunits of the PSI-LHCI supercomplex was performed by uniform labeling of PSI and LHCI subunits with ^14^C and subsequent separation of the polypeptides by three different SDS-PAGE systems. Since the PSI-LHCI supercomplex binds approximately two copies of LHCA1 and one copy of each of the other eight LHCI subunits, it was concluded that 10 LHCI subunits are present in this particular PSI-LHCI supercomplex. Subsequently, we determined the full configuration of the 10 LHCI subunits in the green algal PSI-LHCI supercomplex by chemical cross-linking, assigning the identities of the cross-linked products by immunodetection and mass spectrometry. These results provide new insights into the LHCI configuration linked to the PSI core, suggesting novel functional implications for LHCA2 and LHCA9. These observations also shed new light on the evolution of light-harvesting systems and PSI, particularly in light of the recent PSI-LHCR structure from the red alga *C. merolae* (Pi et al., 2018).

## MATERIALS AND METHODS

### Strains and Growth Conditions

*Chlamydomonas reinhardtii* cells from wild-type strain 137C (for the identification of cross-linked protein via LC-MS/MS analysis, we used strain 4a+ [Peers et al., 2009]) and Δ*psaA*/*B* (Redding et al., 1998), Δ*LHCA2* (LMJ.RY0402.109691), and Δ*LHCA5* (LMJ.RY0402.044057) mutants and were grown to midlog phase (2 to 5 × 10^6^ cells mL^−1^) at 25°C in TAP medium under 20 to 50 μmol photons m^−2^ s^−1^ or in HSM medium under 100 μmol photons m^−2^ s^−1^. For the identification of cross-linked proteins via LC-MS/MS, 4a+ cells pregrown in TAP medium with continuous low light (20–40 μE m^−2^ s^−1^) to midlog phase (1 × 10^6^ cells mL^−1^) were harvested and resuspended to 1 × 10^6^ cells mL^−1^ in HSM and subsequently grown under high light (∼185 μE m^−2^ s^−1^) for 24 h.

### Genetic Characterization of Δ*LHCA2* and Δ*LHCA5* Mutants

The two *C*. *reinhardtii* mutants, Δ*LHCA2* (LMJ.RY0402.109691) and Δ*LHCA5* (LMJ.RY0402.044057), were obtained through the *Chlamydomonas* Resource Center (Li et al., 2016). The insertion sites of the CIB1 cassette were determined by PCR and DNA sequencing using total cellular DNA isolated as described (Kuroda et al., 2014) as a template and the primers as shown (Li et al., 2016).

### Uniform Radiolabeling of Cellular Proteins with ^14^C

Uniform radiolabeling of cellular proteins with ^14^C and isolation of thylakoid membranes and the PSI-LHCI supercomplex were performed as described previously with some modifications (Ozawa et al., 2010; Takahashi et al., 2014). Wild-type cells grown in HSM medium were grown in the presence of 2 μCi mL^−1^ [^14^C]sodium acetate (GE Healthcare) for 24 to 36 h to uniformly radiolabel the total cellular proteins. The thylakoid membranes were isolated, washed with 2 m NaBr to remove extrinsic proteins, and solubilized with 0.8% (w/v) β-DDM. PSI-LHCI was purified from the thylakoid extracts by sucrose density gradient ultracentrifugation (0.1–1.3 m sucrose gradient containing 0.05% (w/v) β-DDM) with a TLS-55 rotor (Beckman Coulter) at 259,000*g* for 2.5 h. The labeled polypeptides were separated by the three different SDS-PAGE systems as described in “Results” and below. Autoradiographs were obtained using an FLA-7000 (Fujifilm) at 25-μm resolution with Imaging Plate (BAS-IP MS2040; Fujifilm), and the signals were quantified with MultiGauge (version 3.0; Fujifilm). Quantification was carried out for three biological replicates.

### PAGE

SDS-PAGE was carried out according to the methods of the High-Molarity-Tris (Fling and Gregerson, 1986) and MES-Tris-Urea (Kashino et al., 2001) systems with some modifications. The High-Molarity-Tris system contained a 15% to 22.5% (w/v) polyacrylamide gradient on the separation gel, and electrophoresis was performed at 6°C or 55°C. The MES-Tris-Urea system contained 18% (w/v) acrylamide and 6 m urea on the separation gel at room temperature.

### BN-PAGE

BN-PAGE was performed as described (Schägger and von Jagow, 1991) with some modifications. A linear 5% to 12% (w/v) acrylamide (2.6% C mixture containing 1.04 g of bisacrylamide per 100 mL) gradient on the separating gel was used. The PSI-LHCI fraction obtained by sucrose density gradient ultracentrifugation was subjected to BN-PAGE. The cathode buffer containing Coomassie Brilliant Blue was replaced with Coomassie Brilliant Blue-free cathode buffer in the midcourse of the electrophoresis to remove excess Coomassie Brilliant Blue from the gel.

### Chemical Cross-Linking

Chemical cross-linking reactions were performed as follows and were quenched by the addition of Tris-HCl (pH 7.5) to a final concentration of 50 mm. Thylakoid membranes (0.2 mg Chl mL^−1^) in 0.1 m MES-NaOH (pH 6) were treated with 2 mm EDC and 5 mm *N*-hydroxysulfosuccinimide at 25°C for 15 min, at which point 2-mercaptoethanol was added to 20 mm and incubation was allowed to proceed at 25°C for another 15 min. Thylakoid membranes (0.2 mg Chl mL^−1^) in 20 mm HEPES-NaOH (pH 8) were treated with 0.5 mm DSS at 25°C for 30 min. Thylakoid membranes (0.8 mg Chl mL^−1^) in 200 mm HEPES-NaOH (pH 8) or PSI-LHCI supercomplexes (0.2 mg Chl mL^−1^) in 200 mm HEPES-NaOH (pH 8) and 0.05% (w/v) β-DDM were treated with 0.5 mm DSG on ice for 2 h. Thylakoid membranes (0.8 mg Chl mL^−1^) in 0.2 m triethanolamine were treated with 5 mm DMS at 25°C for 30 min.

To identify chemically cross-linked polypeptides by LC-MS/MS, thylakoid membranes were incubated with DSS, from which PSI complexes were purified prior to tryptic digestion. An aliquot (200 μg) of thylakoid membranes, isolated as described previously (Bergner et al., 2015), was cross-linked with isotopically labeled DSS, DSS H12/D12 (Creative Molecules) and then solubilized with 1% (w/v) α-DDM; subsequently, PSI-LHCI was recovered as described previously (Tokutsu et al., 2012; Xue et al., 2015), polypeptides of which were separated by SDS-PAGE and stained with Coomassie Brilliant Blue. Gel pieces were excised, and polypeptides were digested with trypsin using the FASP protocol (Wiśniewski et al., 2009). The peptides obtained were subjected to LC-MS/MS analysis.

### Purification of PSI-LHCI Complexes from the Wild Type and Δ*LHCA2* and Δ*LHCA5* Mutants

Thylakoid membranes (0.8 mg Chl mL^−1^) were isolated from wild-type, Δ*LHCA2*, and Δ*LHCA5* cells grown to midlog phase (2 to 4 × 10^6^ cells mL^−1^) as described previously (Ozawa et al., 2010) and solubilized with 1% (w/v) β-DDM. The resulting thylakoid extracts were subjected to sucrose density gradient (0.1–1.3 m Suc gradient containing 0.05% (w/v) β-DDM) ultracentrifugation (Beckman; SW41Ti at 288,000*g* at 4°C for 16 h), and the PSI-LHCI complexes were fractionated and analyzed.

### Purification of the PSI Core Complex Associating with LHCA2 and LHCA9

Thylakoid membranes (0.8 mg Chl mL^−1^) washed with 2 m NaBr were first solubilized with 0.4% (w/v) *n*-tridecyl-β-d-maltoside and then separated by sucrose density gradient (0.4–1.3 m sucrose containing 0.005% (w/v) *n*-tridecyl-β-d-maltoside) ultracentrifugation (Beckman; SW32Ti at 32,000 rpm at 4°C for 20 h). Band A4 was collected (Supplemental Fig. S5A, a2), diluted with 1.5 volumes of buffer (5 mm HEPES-NaOH, pH 8, 10 mm EDTA-NaOH, pH 8, and 0.02% (w/v) β-DDM), solubilized with 0.8% (w/v) β-DDM, and subjected to a second round of sucrose density gradient (0.4–1.3 m sucrose containing 0.02% (w/v) β-DDM) ultracentrifugation (Beckman; SW32Ti at 32,000 rpm at 4°C for 17 h). Band B4 was collected (Supplemental Figure S5A, b), solubilized with 0.8% (w/v) β-DDM, subjected to ion-exchange column chromatography (DEAE Toyopearl 650S; Tosoh) as described (Ozawa et al., 2010), and eluted with buffer (20 mm Tricine-NaOH, pH 8 and 0.02% (w/v) β-DDM) containing a linear gradient of NaCl (0–200 mm) at 4°C (Supplemental Fig. S5B). Column chromatography was performed with a BioLogic LP system (Bio-Rad). Size-exclusion column chromatography was performed with two tandemly connected Superose 6 HR 30 columns (Supplemental Fig. S5C) using an ÄKTAexplorer 10S (GE Healthcare), according to the previously reported method (Ozawa et al., 2010).

### Purification of LHCI

LHCI was purified as described (Takahashi et al., 2004) with some modifications. Thylakoid membrane (0.8 mg Chl mL^−1^) isolated from the Δ*psaA*/*B* mutant was washed with 2 m NaBr and solubilized with 1% (w/v) β-DDM, and the LHCI-enriched fraction was obtained by sucrose density gradient ultracentrifugation. The LHCI complex was purified further by ion-exchange column chromatography with a DEAE Toyopearl PAK 650S (Tosoh) and was eluted with buffer (50 mm Tris-HCl, pH 8, and 0.05% (w/v) β-DDM) containing a linear NaCl concentration gradient of 0 to 200 mm at 4°C using an ÄKTAexplorer 10S (GE Healthcare).

### Immunoblotting, Protein Staining, and N-Terminal Amino Acid Sequencing

Immunoblotting was performed as described previously (Ozawa et al., 2009). Antibodies against LHCA1, LHCA2, LHCA5, LHCA7, LHCA8, PSAH, PSAL, PSAN, and PSAO were generated in rabbits using synthesized oligopeptides, CKATWFGIEVPFD, CEATKTLNPGKESV, CHSVDVQGLTIPLT, CAAVRPVWFPGNPPPA, CEASLKGTSE, CKYGENSRYFDLQD, CKTLSGRSVARD, CNDKKRLATSYAN, and CKSFPRDWVKTD, respectively (Sigma Genosys). Antibody against LHCA3 was described previously (Hippler et al., 2001), and antibodies against LHCA4 and LHCA9 were generated using synthesized peptides (CAVPENVKEREWIDAW and CARPWLPGLNPPAHLK, respectively; Eurogentec). The carrier protein, keyhole limpet hemocyanin, was conjugated to each oligopeptide at the N-terminal Cys residue. An antibody against LHCA6 was generated using the partial amino acid sequence of LHCA6 overexpressed in bacteria. We amplified a portion of the coding region (codons 102–154) of LHCA6 by PCR using total cellular DNA as a template and a pair of oligonucleotides, 5′-GAAGGAGATATA*CATATG*AAGGAGGTTGAGTCG-3′ and 5′-**GTGGTGGTGGTGGTG**CACCTCGTGGGGGGGCA-3′ [where the pET-22b(+) sequence is underlined, the *Nde*I restriction site is in italics, and the 6×His encoding sequence is in boldface]. The amplified fragment was cloned into a pET-22b(+) vector at the *Nde*I site using the In-Fusion technique (Clontech-Takara); the resulting construct encoded a fragment of LHCA6 with a C-terminal 6×His tag. The overexpressed protein was purified by Ni-NTA agarose affinity column chromatography (Qiagen) and subsequent preparative SDS-PAGE. The excised gel slices were homogenized and injected into rabbits (Japan Bioserum).

Immunoblotting signals were detected by enhanced chemiluminescence using an LAS-4000 mini Luminoimage analyzer (Fujifilm) and were quantified with MultiGauge software (version 3.0; Fujifilm). Polypeptides separated by SDS-PAGE were stained with Flamingo fluorescent dye (Bio-Rad); signals were detected with an FLA-7000 Fluoroimage analyzer (Fujifilm) and quantified with MultiGauge. N-terminal amino acid sequencing of proteins blotted onto PVDF filters was carried out using a PPSQ-31A protein sequencer (Shimadzu) as described (Ozawa et al., 2009).

### Pigment and Spectroscopic Analyses

Pigments were extracted using *N*,*N*-dimethylformamide and analyzed by reverse-phase column chromatography as described (Ozawa et al., 2012). Absorption spectra (Supplemental Fig. S5C, c) were measured with a double-beam spectrophotometer (UV2450S; Shimadzu). Fluorescence emission spectra (Supplemental Fig. S5C, d) were measured at 77K as described (Takahashi et al., 2004) using an F-7000 fluorescence spectrophotometer (Hitachi). Samples were excited with an actinic light at 430 nm.

### Mass Spectrometry for Identification of Cross-Linked Proteins

LC-MS/MS analysis was performed as described previously (Hochmal et al., 2016) with some modifications. The flow rate for peptide elution from the separation column was altered to 300 mL min^−1^ for the following gradient profile: 2.5% to 7.5% (v/v) B over 4 min, 7.5% (v/v) to 40% Bover 21 min, 40% (v/v) B over 3 min, 40% to 99% (v/v) B over 3 min, and 99% (v/v) B for 10 min. Afterwards, the column was reequilibrated with 97.5% A for 25 min. A Q Exactive Plus mass spectrometer (Thermo Scientific) was used with the following changes: MS/MS data were acquired in a data-dependent manner, dynamically choosing the 12 most abundant precursor ions from the survey scans. Each sample was measured twice, once without mass tags and once with mass tags enabled. The mass tags corresponded to the H12/D12 difference from the DSS cross-linker with 12.07532 Da and the charge states that were allowed for fragmentation. Dynamic exclusion was enabled, with an exclusion duration of 30 s. The scan ranges were *m/z* 400 to 2,000 for survey scans and *m/z* 200 to 2,000 for fragmentation scans (MS/MS). The AGC target value for MS/MS was 1e5 at 100 ms maximum injection time. The resolution for higher energy C-trap dissociation spectra was set to 35,000. Singly and doubly charged ions, ions above charge state 8, as well as ions with unassigned charge states were excluded from fragmentation. Internal lock mass calibration on *m/z* 445.120025 was enabled. Raw files were converted to mzML files using Proteome Discoverer (version 1.4; Thermo Scientific). mzML files for use with Kojak were converted with ProteoWizard (version 3.0.7389; Chambers et al., 2012).

### Evaluation of MS/MS Data for Identification of Cross-Linked Proteins

MS/MS data were evaluated with the tools described previously (Höhner et al., 2013). Database searches for non-cross-linked peptides were conducted as described (Höhner et al., 2013) with the following changes. Open mass spectrometry search algorithm (OMSSA; Geer et al., 2004) and X! Tandem (version 2013.09.01; Craig and Beavis, 2004) were used, and the *C. reinhardtii* proteome was JGI version 5.5. The mass tolerances were set to 10 ppm for precursor ions and 0.05 Da/10 ppm for product ions, oxidation of Met was set as the only variable modification, and the maximum precursor ion charge was set to 6. For qvality (Käll et al., 2009), the posterior error probability was set at 0.01.

### Crosslinx

Database searches for cross-linked peptides were conducted with Crosslinx (version 0.7.1, gitlab.com/bald/crosslinx). pymzML (Bald et al., 2012) is employed for very fast access to mzML files and provides handling methods for spectra such as deconvolution of MS2 spectra (calculation of masses from *m/z* values). As Crosslinx is able to use deconvolution on high-mass-accuracy MS2 spectra, it only needs to calculate in silico masses for fragment ions instead of all possible *m/z* values, thereby gaining speed. Crosslinx scores hits with an E-value based on a classic probability score as described for normal peptides (Geer et al., 2004). Crosslinx overcomes the limitations of other tools as it is available for several operating systems (Windows, Linux, and Mac), execution times, and the maximum number of spectra or proteins that can be analyzed. Supplemental Table S1 shows that most Crosslinx hits also are found with other tools, such as StavroX (Götze et al., 2012) and Kojak (Hoopmann et al., 2015). Crosslinx is employed in two steps: an initial search against a small database and a validation search against the whole proteome, as described in Supplemental Figure S2. Crosslinx is available for several operating systems, both at the command line interface and with a graphical user interface. Crosslinx is open-source software and can be downloaded at gitlab.com/bald/crosslinx.

### Mapping Cross-Links to Molecular Structure

The identified cross-links were mapped to the molecular structures of the corresponding proteins if structural data were available. For PSI proteins, the high-resolution structure from vascular plants was used (protein database identifier 4Y28). If the cross-linked peptide could not be mapped directly onto the vascular plant sequence, the position of the cross-linked amino acid was estimated by performing a BLAST search (standard parameters for BLASTP adjusted for a short input sequence) with the cross-linked peptide as the query and the sequence from the structure as the subject sequence. For LHCA2, the cross-linked amino acid position could not be estimated by this approach; therefore, Phyre2 (Kelley et al., 2015) was used to model the protein based on the *C*. *reinhardtii* protein sequence. Using PyMOL (The PyMOL Molecular Graphics System, version 1.8; Schrödinger), the modeled LHCA2 was aligned to LHCA2 from vascular plants, rotated by approximately 90°, and shifted in the membrane plane to the PSAH side of the photosystem core until an LHCA2 position was reached where all cross-link distance restraints (less than 30 Å; Merkley et al., 2014) were fulfilled. The distances were calculated as the C_α_ distances of the cross-linked amino acids using PyMOL to check for the distance restraints of 26 to 30 Å (Merkley et al., 2014). Molecular representations were created with PyMOL.

### Accession Numbers

Accession numbers (UniProt Knowledgebase) are as follows: PSAA, P12154; PSAB, P09144; PSAC, Q00914; PSAD, Q5NKW4; PSAE, A8ICV4; PSAF, A8J4S1; PSAG, P14224; PSAH, A8IH77; PSAI, A8IFG7; PSAJ, P59777; PSAK, A8J6K8; PSAL, A8IL32; PSAN, Q9AXJ2; PSAO, A8JCL6; LHCA1, Q05093; LHCA2, A8IKC8; LHCA3, Q75VY9; LHCA4, Q75VZ0; LHCA5, Q75VY8; LHCA6, Q75VY6; LHCA7, Q84Y02; LHCA8, Q75VY7; and LHCA9, A8ITV3.

### Supplemental Data

The following supplemental materials are available.

**Supplemental Figure S1.** Effect of growth medium on LHCI accumulation of the PSI-LHCI supercomplex.
**Supplemental Figure S2.** Crosslinx evaluation strategy for MS/MS data from cross-linked samples.
**Supplemental Figure S3.** Adjacency relationships among the cross-linked subunits.
**Supplemental Figure S4.** Genetic characterization of Δ*LHCA2* and Δ*LHCA5* mutants.
**Supplemental Figure S5.** Isolation of PSI-LHCI subcomplexes retaining LHCA2 and LHCA9.
**Supplemental Figure S6.** Oligomeric LHCI complex from a PSI-deficient mutant.
**Supplemental Table S1.** Characteristics of the nine LHCI subunits.
**Supplemental Table S2.** Cross-linked PSI proteins identified with Crosslinx in MS/MS data from DSSd0/d12 cross-linked *C. reinhardtii* thylakoids.
**Supplemental Data Set S1.** All cross-linked peptide/spectral matches.
**Supplemental Data Set S2.** Spectrum plots of all verified cross-linked peptide/spectral matches, generated with pymzML.

## Dive Curated Terms

The following phenotypic, genotypic, and functional terms are of significance to the work described in this paper:

CIB1 Gramene: AT4G34530
CIB1 Araport: AT4G34530

LHCA1 Gramene: AT3G54890
LHCA1 Araport: AT3G54890

LHCA3 Gramene: AT1G61520
LHCA3 Araport: AT1G61520

LHCA4 Gramene: AT3G47470
LHCA4 Araport: AT3G47470

LHCA6 Gramene: AT1G19150
LHCA6 Araport: AT1G19150

psaA Gramene: ATCT00350
psaA Araport: ATCT00350

PSAF Gramene: AT1G31330
PSAF Araport: AT1G31330

PSAG Gramene: AT1G55670
PSAG Araport: AT1G55670

PsaJ Gramene: ATCT00630
PsaJ Araport: ATCT00630

PsaK Gramene: AT1G30380
PsaK Araport: AT1G30380

PSAL Gramene: AT4G12800
PSAL Araport: AT4G12800

PSAN Gramene: AT5G64040
PSAN Araport: AT5G64040

PSAO Gramene: AT1G08380
PSAO Araport: AT1G08380

polypeptides CHEBI: CHEBI:15841
